# Socioeconomic and geographical inequalities in health care coverage in Mozambique: a repeated cross-sectional study of the 2015 and 2018 national surveys

**DOI:** 10.1186/s12889-023-15988-y

**Published:** 2023-05-30

**Authors:** Chanvo S. L. Daca, Miguel San Sebastian, Carlos Arnaldo, Barbara Schumann, Fredinah Namatovu

**Affiliations:** 1 Directorate of Planning and Cooperation, Ministry of Health, Maputo, Mozambique; 2 Department of Epidemiology and Global Health, Umeå University, Umeå, Sweden; 3 Centre for African Studies, Universidade Eduardo Mondlane, Maputo, Mozambique; 4 Department of Health and Caring Sciences, Linnaeus University, Kalmar, Sweden

**Keywords:** Health care coverage, Health inequality, National surveys, Socioeconomic inequalities, Mozambique

## Abstract

**Background:**

Over the past years, Mozambique has implemented several initiatives to ensure equitable coverage to health care services. While there have been some achievements in health care coverage at the population level, the effects of these initiatives on social inequalities have not been analysed.

**Objective:**

The present study aimed to assess changes in socioeconomic and geographical inequalities (education, wealth, region, place of residence) in health care coverage between 2015 and 2018 in Mozambique.

**Methods:**

The study was based on repeated cross-sectional surveys from nationally representative samples: the Survey of Indicators on Immunisation, Malaria and HIV/AIDS in Mozambique (IMASIDA) 2015 and the 2018 Malaria Indicator survey. Data from women of reproductive age (15 to 49 years) were analysed to evaluate health care coverage of three indicators: insecticide-treated net use, fever treatment of children, and use of Fansidar malaria prophylaxis for pregnant women. Absolute risk differences and the slope index of inequality (SII) were calculated for the 2015 survey period and the 2018 survey period, respectively. An interaction term between the socioeconomic and geographical variables and the period was included to assess inequality changes between 2015 and 2018.

**Results:**

The non-use of insecticide-treated nets dropped, whereas the proportion of women with children who were not treated for fever and the prevalence of women who did not take the full Fansidar dose during pregnancy decreased between 2015 and 2018. Significant reductions in the inequality related to insecticide-treated net use were observed for all socioeconomic variables. Concerning fever treatment, some reductions in socioeconomic inequalities were observed, though not statistically significant. For malaria prophylaxis, the SII was significant for education, wealth, and residence in both periods, but no significant inequality reductions were observed in any of these variables over time.

**Conclusions:**

We observed significant reductions of socioeconomic inequalities in insecticide-treated net use, but not in fever treatment of children and Fansidar prophylaxis for pregnant women. Decision-makers should target underserved populations, specifically the non-educated, poor, and rural women, to address inequalities in health care coverage.

## Background

The association between socioeconomic status and health as well as health care coverage has been widely investigated [1–5]. In the sub-Saharan African context, a systematic review revealed that low education and poverty were associated with increased risk of malaria infection [6]. Another multi-country study found that poor women lacked maternal health care services in Burkina Faso and Senegal. Investment to foster social and economic development through innovations from multiple sectors has benefitted lagging regions and reduced under-five mortality in Ethiopia [7]. Investing in socially disadvantaged groups through sound policies has the potential to trigger health benefits for all social groups, specifically by increasing coverage to health care and health-promotion programmes [8].

Mozambique is a low-income country located in the southeast African region, with a Gini index of 54% in 2014 [9]. Over the past years, the country has implemented several plans and strategies aiming to improve population health and to decrease socioeconomic inequalities in health. In 2014, the Ministry of Health (MoH) introduced the Health Sector Strategic Plan 2014–2019, which established a series of strategic objectives that included increasing coverage and utilisation of health services, improving the quality of service, and reducing geographical and social inequities. In 2017, the government additionally launched, with support from international donors, a comprehensive programme in reproductive, maternal, neonatal, child, and adolescent health and nutrition, with a focus on reducing social inequalities in coverage of these services [10]. Available data suggest that some health and health service coverage indicators have improved over the past years [11, 12]. However, while the reduction of social inequalities in health has been the focus of several public health interventions in the country, to the best of our knowledge, no study has been conducted to assess if these inequalities have changed over time.

The aim of this study was to assess whether socioeconomic and geographical inequalities in women’s and children’s health care coverage (inequalities in bed net use, Fansidar prophylaxis, and fever treatment) decreased in Mozambique during the 2015–2018 period.

## Methods

### Study design and data source

This was a repeated cross-sectional study that used the Survey of Indicators on Immunisation, Malaria and HIV/AIDS in Mozambique (IMASIDA) 2015 and the 2018 Malaria Indicator survey (MIS). The surveys consist of four questionnaires: the household, biomarkers, and a survey for men and women each. For this study, only the women’s questionnaire was used.

### Population and Sample

The Demographic and Health Survey (DHS) Programme conducted both surveys using nationally representative samples of 6,964 and 6,279 households in 2015 and 2018, respectively.

The DHS follows a two-stage probability sample design drawn from the most recent census, stratified by geographic province and by urban/rural areas within each province. The primary sampling units were the census enumeration areas, forming the survey clusters. In the second stage, a complete household listing was conducted in each of the selected clusters.

### Outcomes variables

Three variables representing different dimensions of health care coverage were selected: the use of insecticide-treated nets (ITNs) for children under five, fever treatment of children under five, and Fansidar malaria prophylaxis for pregnant women. These variables were selected because of the following reasons: (i) to maintain consistency, since this work is part of a bigger research project focused on maternal and child health where similar outcomes were used; (ii) the variables are part of the WHO framework that monitors universal health coverage [13]; (iii) they were available in both surveys and (iv) they capture different aspects of health service coverage.

The ITN subsample consisted of 4,756 women with children under five in 2015 and 4,204 in 2018. The fever treatment sub-sample consisted of 1,134 and 1,144 women in 2015 and 2018, respectively. For Fansidar prophylaxis, this sub-sample consisted of 3,977 women in 2015 and 3,524 in 2018.

The ITNs are freely distributed for target groups (women and children) at antenatal care services or through communities’ health campaigns. Fever treatment and Fansidar prophylaxis are also provided free of charge at district hospitals (level II) and health posts and health centres (level I) according to need [14]. The use of ITNs was defined by asking a woman with children less than five years old if the child or children had slept under an ITN the night before the survey. Lack of ITN use was categorised as “yes” if at least one child had not slept under the bed net.

Lack of fever treatment — a standard treatment for children of a certain age (indicated for a range of common childhood diseases, e.g., malaria) — was assessed by asking women with a child under the age of five if the child had had a fever two weeks before the survey. If the answer was affirmative, the follow-up question was if she had sought counselling or fever treatment. Lack of treatment was then dichotomised into “no” and “yes”.

In Mozambique, the national antenatal care guidelines recommend that pregnant women take three doses of Fansidar to prevent malaria infection [15]. Women were asked: “During the pregnancy of your last child, did you take Fansidar to prevent malaria?” If the answer was yes, then the follow-up question was how many times. If any of the recommended doses could not be verified by the antenatal card or reported by the mother, then the participant was classified as not having received the recommended doses.

### Socioeconomic and geographical variables

The independent variables, based on the data available in both surveys, included age, education, wealth, place of residence, and region. Age of the mother was categorised into three groups: 15 to 24, 25 to 39, and 40 to 49 years. Education was classified into three categories: no education, completed primary school, and completed secondary school, or higher than secondary. Wealth index, a proxy for household income, was calculated by the DHS program based on the following households´ assets: television; car; and dwelling characteristics such as flooring material, type of drinking water source, and toilet facilities [16]. Place of residence was dichotomised into rural or urban residence, and the 11 administrative provinces were grouped into three regions: northern (Niassa, Cabo Delgado, and Nampula), central (Zambezia, Sofala, Manica, and Tete), and southern (Maputo, Gaza, and Inhambane). Urban and the southern region were used as reference categories since they are considered as more socially advantaged areas [17] .

### Data analysis

The population characteristics were summarised using descriptive statistics to calculate the prevalence of each of the health care outcomes in 2015 and 2018. Then absolute risk differences (ARDs) were estimated as the measure of association between the socioeconomic variables and the outcomes. In addition, the slope index of inequality (SII) was calculated to obtain the absolute social gradient in the outcomes [18]. The SII is a weighted summary measure of health inequality that represents the absolute difference in the estimated values of a health indicator between the most and the least advantaged group, while taking into consideration the population distribution across the social categories [19]. To estimate the SII, *ridit* scores, corresponding to the mid-point of the average cumulative proportion of the population in each category of the socio-economic variable, were first calculated [20]. Then, SII coefficients were obtained by generalised linear models with the outcome regressed on the *ridit* scores, separately for each socioeconomic indicator. If there is no inequality, the SII takes the value of zero, while other values indicate social inequality in health. In this study, positive values indicated higher use of the outcome in the socially advantaged subgroups, while negative values indicated higher use in the disadvantaged subgroups. Finally, an interaction term between the *ridit* scores of the socio-economic variables and time period was included to calculate the SII difference that quantifies and tests the statistical significance of changes in socioeconomic inequalities between 2015 and 2018 [21]. Analyses considered the two-stage probability design of the surveys (using the Stata svyset command), the weighting procedure (as recommended by the DHS Program) and were adjusted for age. All regression models were estimated using a generalized linear model with a binomial distribution and the 95% confidence intervals of the ARD, SII, and SII differences were used to express statistical inference. If the interval did not include zero, the difference was considered to be statistically significant. The analyses were conducted using Stata 15.1 statistical software [22].

### Ethical clearance

For this study, IMASIDA and MIS data were obtained from the DHS website [http://www.measuredhs.com]. These data are anonymous and publicly available; their usage is covered by the ethical approval secured by the DHS for the data collection. The IMASIDA and MIS informed consent forms provide details that participation is voluntary, that the respondent may refuse to answer any question or terminate participation at any time, and that the respondent’s identity and information will be kept strictly confidential. Before each interview, the informed consent statement was handed to the respondent, who could accept or decline to participate.

## Results

### Population characteristics

Table 1 shows the weighted prevalence of the socioeconomic and geographical characteristics of the total sample and the outcomes in the two periods. Improvements in education over time were noticed. The percentage of women with no education slightly decreased from 69.52% to 2015 to 62.98% in 2018, whereas the proportion of women with secondary education, albeit still low, nearly doubled in the same period from 5.35 to 9.09%. No significant change was observed in the proportion of people living in the rural region from 2015 (64.76%) to 2018 (64.17%).

**Table 1:** Weighted prevalence of socioeconomic and geographical characteristics and health preventive outcomes of the study population (2015 and 2018) *

	2015		2018	
	N = 6915	**%**	N = 6184	%
**Age group**				
15–24	2,874	41.57	2,624	42.44
25–39	2,838	41.04	2,453	39.68
40–49	1,202	17.39	1,106	17.89
**Education**				
Completed Secondary School or higher than secondary	369	5.35	562	9.09
Completed Primary School	1,738	25.13	1,726	27.93
No education	4,807	69.52	3,895	62.98
**Wealth Quintile**				
Richest	975	14.09	913	14.77
Richer	1,063	15.38	963	15.59
Middle	1,275	18.44	1,214	19.63
Poorer	1,696	24.52	1,481	23.96
Poorest	1,906	27.56	1,611	26.05
**Residence**				
Urban	2,437	35.24	2,216	35.83
Rural	4,478	64.76	3,968	64.17
**Region**				
Northern	2,442	35.31	2,044	33.07
Central	2,502	36.18	2,536	41.01
Southern	1,971	28.51	1,603	25.93
**Outcomes**				
ITN Total sample	4756	100	4204	100
Non-ITN use	2425	51.01	990	23.55
Fever treatment total sample	1134	100	1144	100
Non-fever treatment	399	35.18	322	28.07
Fansidar total sample	3977	100	3524	100
Non-use of 3 Fansidar doses	3117	78.36	2126	60.33

*Numbers weighted for national representation as recommended by DHS population-based random sample selection procedures and the different socioeconomic groups

Table 1 shows a decrease over time in all three outcomes. The proportion of women reporting a child who did not sleep under an ITN dropped by more than half, from 51.01% to 2015 to 23.55% in 2018. The proportion of women with children who were not treated for fever decreased from 35.18% to 2015 to 28.07% in 2018, whereas the prevalence of women who did not take the full Fansidar dose during pregnancy also diminished from 78.36% to 2015 to 60.33% in 2018.

### Socioeconomic and geographical inequalities in health care coverage

Table 2 presents the prevalence, ARD, and SII for lack of ITN use for children. Lack of ITN use was higher in women with no education compared to those with secondary education, in the poorest compared to richest women. Lack of ITN use was highest among those in the middle wealth quintile followed by those in the poorest quintile and in rural compared to urban women. In 2018, on the other hand, the SII was significantly positive only for region, indicating higher lack of ITN use in the northern region compared to the southern region. Between 2015 and 2018, significant reductions in socioeconomic inequalities were observed in all examined variables: education (SII difference = -16.74; 95% CI: -31.16, -2.32), wealth quintile (SII difference = -15.24; 95% CI: -28.60, -1.89), residence (SII difference = -28.03; 95% CI: -46.44, -9.62), and region (SII difference = -23.01; 95% CI: -38.27, -7.75).

**Table 2:** Lack of ITN use: Prevalence, absolute risk difference and slope index of inequality by socioeconomic and geographical factors, 2015 and 2018

	Prevalence		Absolute Risk Difference		Slope Index of Inequality difference
Variable	2015	2018	2015	2018	
Education					
Completed Secondary School or higher than secondary	35.25	17.98	Ref	Ref	
Completed Primary School	44.07	22.09	**9.03 (1.14, 16.92)**	4.07 (-2.76, 10.90)	
No Education	54.31	24.73	**18.82 (11.04, 26.61)**	6.77 (-2.36, 15.91)	
SII			**23.63 (14.95, 32.31)**	7.77 (-4.16, 19.71)	**-16.74 (-31.16, -2.32)**
**Wealth Quintile**					
Richest	42.80	27.96	Ref	Ref	
Richer	44.31	22.07	1.41 (-6.17, 8.99)	-5.87 (-13.56, 1.81)	
Middle	54.37	22.41	**11.34 (3.44, 19.23)**	-5.56 (-14.06, 2.93)	
Poorer	53.13	22.70	**9.98 (2.87, 17.09)**	-5.26 (-13.66, 3.14)	
Poorest	53.51	24.14	**10.41 (3.28, 17.55)**	-3.83 (-12.33, 4.67)	
SII			**13.08 (4.37, 21.80)**	-1.80 (-11.24, 7.63)	**-15.24 (-28.60, -1.89)**
**Residence**					
Urban	43.39	25.50	Ref	Ref	
Rural	54.54	22.63	**10.92 (5.23, 16.60)**	-2.90 (-9.74, 3.93)	
SII			**21.84 (10.46, 33.21)**	-5.81 (-19.49, 7.86)	**-28.03 (-46.44, -9.62)**
**Region**					
Southern	53.84	35.56	Ref	Ref.	
Northern	43.93	18.78	**-10.01 (-17.10, -2.91)**	**-16.87 (-24.61, -9.13)**	
Central	55.53	21.04	1.73 (-4.73, 8.20)	-14.57 (-20.67, 8.47)	
SII			4.85 (-5.67, 15.38)	-**18.27 (-27.73, -8.80)**	**-23.01 (-38.27, -7.75)**

SII - Slope Index of Inequality

SII difference quantifies changes and their statistical significance of socioeconomic inequalities in ITN use between 2015 and 2018

Bold figures: Statistically significant results

All analyses were adjusted for age

Table 3 shows results for lack of fever treatment of children. In 2015, a lower coverage in the socially disadvantaged subgroups was observed, which was statistically significant for education, wealth quintile, and residence. In 2018, only education and wealth quintile remained statically significantly associated with lack of fever treatment. Minor changes between 2015 and 2018, not statistically significant, were noticed in education (SII difference = -7.05; 95% CI: -27.60, 13.48) and wealth quintile (SII difference = **-**0.41; 95% CI: -19.38, 18.55). Finally, despite considerable inequality decreases between regions (SII difference = -20.94; 95% CI: -46.25, 4.36) and residence areas (SII difference = -14.33; 95% CI: -39.93, 11.26), none of these changes were statistically significant.

**Table 3:** Lack of fever treatment: Prevalence, absolute risk difference and slope index of inequality by socioeconomic and geographical factors, 2015 and 2018

	Prevalence		Absolute Risk Difference		Slope Index of Inequality difference
Variable	2015	2018	2015	2018	
Education					
Completed Secondary School or higher than secondary	24.59	31.42	Ref	Ref	
Completed Primary School	23.05	15.07	1.25 (-1.52, 17.76)	-17.30 (-46.28, 11.67)	
No Education	38.61	32.17	13.44 (-2.68, 29.58)	-1.99 (-28.93, 24.95)	
SII			**25.23 (10.22, 40.23)**	**19.27 (3.20, 35.33)**	-7.05 (-27.60, 13.48)
**Wealth Quintile**					
Richest	31.76	18.63	Ref	Ref	
Richer	15.12	13.75	-**16.56 (-27.32, -5.80)**	-5.64 (-14.81, 3.52)	
Middle	33.20	25.91	-00.12 (-11.98 ,11.73)	7.77 (-4.45, 20.00)	
Poorer	33.66	26.38	00.40 (-10.99, 11.80)	6.98 (-3.26, 17.23)	
Poorest	43.51	35.82	10.31 (-00.93, 21.56)	**15.90 (3.48, 28.33)**	
SII			**23.15 (11.56, 34.74)**	**22.87 (9.16, 36.59)**	-0.41 (-19.38, 18.55)
**Residence**					
Urban	23.41	22.16	Ref	Ref	
Rural	38.49	30.08	**13.94 (7.00, 20.87)**	7.13 (-3.13, 17.40)	
SII			**27.88 (14.01, 41.74)**	14.27 (-6.27, 34.81)	-14.33 (-39.93 ,11.26)
**Region**					
Southern	27.81	20.05	Ref	Ref.	
Northern	37.62	38.60	9.09 (-0.03, 18.54)	**16.62 (5.83, 27.42)**	
Central	36.04	22.36	8.20 (-0.05, 16.94)	2.08 (-6.93, 11.10)	
SII			11.07 (-3.98, 26.14)	-8.04 (-26.37, 10.27)	-20.94 (-46.25, 4.36)

SII- Slope index of Inequality

SII difference quantifies changes and their statistical significance of socioeconomic inequalities in fever treatment between 2015 and 2018

Bold figures: - statistically significant results

All analyses were adjusted for age

According to the Table 4, similarly to the other two outcomes, a decrease in the lack of Fansidar prophylaxis use during pregnancy was reported, more pronounced in the socially advantage groups than in the socially vulnerable groups, in both 2015 and 2018, with a statistically significant SII for education, wealth quintile, and residence. However, no significant inequality reductions over time were observed in any of the variables explored: education (SII difference = 7.03; 95% CI: -7.13, 21.21), wealth quintile (SII difference = 6.16; 95% CI: -7.69, 20.02), residence (SII difference = 1.56; 95% CI: -16.31, 19.44), and region (SII difference = 2.68; 95% CI: -13.26, 18.63).

**Table 4:** Lack of Fansidar prophylaxis during pregnancy: Prevalence, absolute risk difference and slope index of inequality by socioeconomic and geographical factors, 2015 and 2018

	Prevalence		Absolute Risk Difference		SII difference
Variable	2015	2018	2015	2018	
Education					
Completed Secondary School or higher than secondary	67.19	47.19	Ref	Ref	
Completed Primary School	74.91	55.34	7.89 (-0.21, 16.01)	**8.71 (1.71, 15.66)**	
No Education	79.98	63.40	**12.68 (3.71, 21.66)**	**16.00 (7.36, 24.65)**	
SII			**12.85 (4.16, 21.54)**	**19.23 (8.38, 30.00)**	7.03 (-7.13, 21.21)
**Wealth Quintile**					
Richest	74.11	43.85	Ref	Ref	
Richer	71.01	52.54	-3.08 (-8.47, 2.30)	8.91 (-2.01, 19.84)	
Middle	71.81	60.54	-2.31 (-8.78, 4.16)	**16.86 (8.66, 25.05)**	
Poorer	83.55	64.67	**9.33 (3.36, 15.29)**	**20.75 (12.74, 28.77)**	
Poorest	81.89	64.26	**7.69 (1.45, 13.94)**	**20.23 (11.46, 29.00)**	
SII			**16.12 (7.99, 24.26)**	**22.08 (11.38, 32.77)**	6.16 (-7.69, 20.02)
**Residence**					
Urban	70.81	52.35	Ref	Ref	
Rural	81.26	63.58	**10.33 (4.27, 16.38)**	**11.00 (4.78, 17.22)**	
SII			**20.66 (8.55, 32.77)**	**22.00 (9.56, 34.45)**	1.56 (-16.31, 19.44)
**Region**					
Southern	77.17	55.43	Ref	Ref.	
Northern	80.63	65.11	3.45 (-2.64, 9.55)	**9.30 (1.03, 17.56)**	
Central	76.94	58.72	-0.02 (-6.06, 5.62)	3.07 (-3.85, 10.01)	
SII			-1.34 (-10.40, 7.71)	1.05 (-10.38, 12.49)	2.68 (-13.26, 18.63)

SII- Slope index of Inequality

SII difference quantifies changes and their statistical significance of socioeconomic inequalities in Fansidar treatment between 2015 and 2018

Bold figures: - statistically significant results

All analyses were adjusted for age

## Discussion

The present study assessed the socioeconomic and geographical inequalities in health care coverage of women and children and how these changed over time in the context of several health initiatives in Mozambique. We observed a higher coverage of ITN, fever treatment, and Fansidar prophylaxis between 2015 and 2018. Our study revealed that the lack of these three outcomes were more prevalent among women with low education, the poorest, and those living in a rural region. We also found a reduction in the socioeconomic inequalities of bed-net coverage, but not for fever treatment and Fansidar prophylaxis, over time. The increase in ITN use in Mozambique could be attributed to efforts of the MoH in implementing diverse malaria preventive measures, as ITNs continue to be an essential component of the national vector control strategies [23]. In 2015, under the Lubombo Spatial Regional Initiative of Mozambique, South Africa, and Swaziland, with developmental partners, ITN distribution was expanded, particularly among disadvantaged populations [24, 25]. Additionally, in 2017 Mozambique started a nationwide ITN distribution with support from the Global Fund, which might have contributed to increased ITN use and reduction of the social inequalities in the country [26]. Though the study still showed a low relative frequency in the use of fever treatment among children and Fansidar prophylaxis in mothers, an overall increase in the two outcomes was observed over time. However, inequalities prevailed: in 2018, the usage was still lower among women with low education, those who were poor, and those living in a rural region. Similar social determinants were shown in previous national studies to be associated with non-treatment of febrile children [27].

Our findings are in line with other sub-Saharan African studies that have linked low Fansidar prophylaxis during pregnancy and infrequent antenatal care visits to low education, poverty, rural residence, and long distance to clinics, among other contextual factors [28]. While approximately 90% of the pregnant women in Mozambique undergo antenatal care, only 55% attend the minimum number of four visits as recommended by the MoH [12]. This lack of continuation might explain the low coverage of Fansidar prophylaxis.

ITNs are distributed at health posts and in health campaigns, which could facilitate their coverage, whereas the provision of fever treatment and Fansidar requires drug provision, laboratory consumables, and qualified health professionals, which are not always available in all health facilities. On the other hand, women, particularly in rural areas, must cope with challenges related to long distances, transportation cost, and different sociocultural barriers in coverage to the health system [29]. In addition, the Mozambican health service sector is facing significant challenges due to a shortage of human resources, deficient management of medical equipment, and lack of laboratory consumables and medicines [30]. Further, the analysis period of our study coincides with a period of political unrest, conflict, and an ongoing debt crisis that might have hindered the health care coverage of disadvantaged groups and the availability of resources at different health system levels. All these factors together might have played a role in the observed persistent health inequalities over time.

### Methodological considerations

The present study includes certain strengths and limitations. The application of the same questionnaires in the two surveys, the availability of data from a large population-based random sample in the two studied periods, as well as the inclusion of several socioeconomic variables increased the validity of the study. In addition, the standard DHS interviewing technique and data collection protocols contributed to minimise reporting bias.

Some limitations should be considered when interpreting the results. Since the observations were self-reported, recall bias could be present. In addition, different factors not assessed in our study such as the sociocultural context and general health care seeking behaviour could be associated with the outcomes. Moreover, the observed changes in inequalities could have been further influenced by different governmental and international programs outside the health sector as well as by social and economic factors that were not captured in this study. Therefore, results should be interpreted with some caution. The study is based on surveys from only two time points; thus, no trend could be studied.

## Conclusions

Overall, this study found increased healthcare coverage among women and children in Mozambique. However, our findings also revealed persistent socioeconomic inequalities for all three analysed outcomes, which were reduced only for ITN use, but not for fever treatment and Fansidar prophylaxis. Several interventions to facilitate the coverage to these health preventive measures are therefore needed to reduce the persistent health inequalities among non-educated, poor, and rural women. Specifically, policy makers should strengthen the existing community health programmes in the country to target underserved populations.

## Data Availability

Requests for the data can be made to the DHS Program at https://dhsprogram.com/what-we-do/survey/survey-display-467.cfm.
